# Loss of AKR1B10 promotes colorectal cancer cells proliferation and migration via regulating FGF1-dependent pathway

**DOI:** 10.18632/aging.103393

**Published:** 2020-07-02

**Authors:** Yizhou Yao, Xuchao Wang, Diyuan Zhou, Hao Li, Huan Qian, Jiawen Zhang, Linhua Jiang, Bin Wang, Qi Lin, Xinguo Zhu

**Affiliations:** 1 Department of General Surgery, The First Affiliated Hospital of Soochow University, Suzhou, Jiangsu, China; 2 Suzhou Emergency Center, Suzhou, Jiangsu, China

**Keywords:** AKR1B10, colorectal cancer, FGF1, targeted therapy

## Abstract

Colorectal cancer (CRC) is a common malignancy worldwide with poor prognosis and survival rates. The aldo-keto reductase family 1 member B10 (AKR1B10) plays an important role in metabolism, cell proliferation and mobility, and is downregulated in CRC. We hypothesized that AKR1B10 would promote CRC genesis via a noncanonical oncogenic pathway and is a novel therapeutic target. In this study, AKR1B10 expression levels in 135 pairs of CRC and para-tumor tissues were examined, and its oncogenic role was determined using *in vitro* and *in vivo* functional assays following genetic manipulation of CRC cells. AKR1B10 was downregulated in CRC tissues compared to the adjacent normal colorectal tissues, and associated with the clinicopathological status of the patients. AKR1B10 depletion promoted the proliferation and migration of CRC cells *in vitro*, while its ectopic expression had the opposite effect. AKR1B10 was also significantly correlated with FGF1 gene and protein levels. Knockdown of AKR1B10 promoted tumor growth *in vivo*, and increased the expression of FGF1. Finally, AKR1B10 inhibited FGF1, and suppressed the proliferation and migration ability of CRC cells in an FGF1-dependent manner. In conclusion, AKR1B10 acts as a tumor suppressor in CRC by inactivating FGF1, and is a novel target for combination therapy of CRC.

## INTRODUCTION

Colorectal cancer (CRC) is one of the most commonly diagnosed malignancies worldwide, and is associated with high morbidity and mortality [1]. Apart from surgical resection, several targeted therapies have been developed against CRC in order to improve prognosis. However, the complex mechanism of CRC genesis considerably limits the therapeutic outcomes in advanced cancer [2, 3]. Therefore, it is essential to determine the mechanisms underlying the development and progression of CRC in order to identity novel therapeutic targets.

Aldo-keto reductase family 1 member B10 (AKR1B10), a member of the AKR1B subfamily, is a 36-kDA cytosolic NADPH-dependent oxidoreductase that catalyzes the reduction of intracellular reactive oxygen species (ROS), retinaldehyde, lipid peroxidation products and xenobiotics [4, 5]. It is commonly expressed in normal epithelial tissues of the digestive tract and presents at very low level in non-gastrointestinal tissues [6, 7]. Aberrant expression of AKR1B10 has been detected in multiple solid tumors such as hepatocellular cancer [8], lung cancer [9], breast cancer [10] and pancreatic cancer [11], and strongly associated with prognosis [12–15], and downregulated in malignancies of the digestive tract, such as gastric cancer and CRC [15, 16]. AKR1B10 normally exerts a gastro-protective effect by metabolizing α, β-unsaturated carbonyl compounds produced by gut microbiota into less toxic hydroxyl compounds [17], promoting the synthesis of fatty acids or lipids in the digestive tract mucosa for the constant renewal of crypt cells [18], and mediating retinoid acid homeostasis and cell differentiation [10]. Thus, it is not surprising that aberrantly low level of AKR1B10 in the gastrointestinal tract is closely linked with the development of cancers [15, 16], as well as inflammatory conditions like diabetic nephropathy [19]. However, little is known regarding the role of AKR1B10 in CRC development, and the molecular mechanisms remain elusive.

Fibroblast growth factor 1 (FGF1) was first identified in brain and pituitary tissues [10], and functions as an insulin sensitizer in type 2 diabetes mellitus along with maintaining adipose tissue and metabolic homeostasis [20, 21]. Studies have also reported anti-inflammatory effects of FGF1 [21, 22], which is significant since metabolic disorders often progress to tumors due to adipose inflammation and systemic circulation of metabolic and inflammatory factors [23]. Therefore, we hypothesized that high level expression of AKR1B10 would suppress CRC development via a non-canonical FGF1-dependent pathway, and our findings demonstrated a novel role of AKR1B10 in CRC and identified its potential diagnostic and therapeutic relevance.

## RESULTS

### AKR1B10 is downregulated in CRC tissues and related to poor prognosis

**Figure 1:**
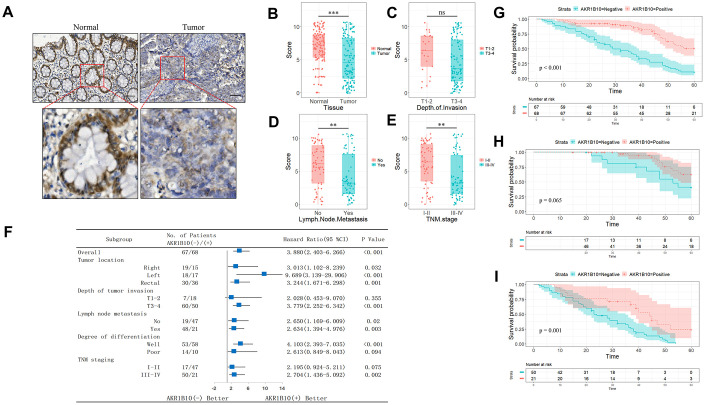
**Expression of AKR1B10 in CRC tissues.** (**A**) Representative IHC images showing *in situ* AKR1B10 expression in CRC and normal tissues (scale bar = 100μm). (**B**–**E**) IHC scores of AKR1B10 in (**B**) CRC vs normal tissues, (**C**) T I-II vs T III-IV tissues, (**D**) tumors with or without lymph node invasion, and (**E**) early vs late TNM staging. (**F**) OS of AKR1B10^pos^ and AKRiB10^neg^ CRC patients in subgroups demarcated by tumor location, depth of tumor invasion, lymph node metastasis, degree of differentiation and TNM staging. (**G**–**I**) OS of (**G**) AKR1B10^pos^ and AKRiB10^neg^ CRC patients with TNM staging I-II (**H**) and III-IV (**I**). CRC, colorectal cancer. OS, overall survival. ns, no significant difference. ** P < 0.01, *** P < 0.001.

The AKR1B10 protein was highly expressed in normal colorectal tissues, and significantly lower in the CRC tissues (Figure 1A–1B, Supplementary Figure 1A). Although *in situ* AKR1B10 levels were similar between the T1-2 and T3-4 tissues (*P* > 0.05; Figure 1C), it was significantly decreased in patients with lymph node invasion compared with those without (*P* < 0.01, Figure 1D). Furthermore, AKR1B10 expression was reduced in CRC tissues with tumor-node-metastasis (TNM) staging I-II compared to III-IV (*P* < 0.01; Figure 1E). Our results were confirmed with TCGA datasets in the GEPIA platform (Supplementary Figure 1B). In addition, AKR1B10 expression was significantly associated with the depth of invasion (*P* < 0.05), lymph node invasion (*P* < 0.001) and TNM staging (*P* < 0.001, Table 1), while no correlation was observed with other clinicopathological variables such as age, gender, tumor size, tumor location or degree of differentiation (*P* > 0.05; Table 1). Univariate analysis further revealed that low AKR1B10 expression (*P* < 0.001), lymph node invasion (*P* < 0.001), degree of differentiation (*P* < 0.01), depth of invasion (P < 0.001) and TNM staging (*P* < 0.001, Table 2) were related to poor prognosis, and low AKR1B10 expression was confirmed as an independent prognostic factor for the survival of CRC patients by multivariate analysis (*P* < 0.001, Table 2). Therefore, we demarcated the patients according to AKR1B10 expression levels, and found that the survival of AKR1B10^neg^ patients was significantly worse compared to the AKR1B10^pos^ group (*P* < 0.05; Figure 1F–1G, Supplementary Figure 1C), regardless of age, gender, tumor size, tumor location, venous invasion, neural invasion and lymph node metastasis. In contrast, AKR1B10 expression level had no bearing on the survival of patients with staging T1-T2 invasion (*P* = 0.355), poor differentiation (*P* = 0.094) and TNM staging I-II (*P* = 0.075). Interestingly, elevated AKR1B10 expression was associated with favorable prognosis in patients with TNM staging III-IV but not the staging I-II patients (*P* = 0.065; *P* = 0.001; Figure 1H–1I).

**Table 1:** Relationship between AKR1B10 and clinic-pathological factors in 135 CRC patients.

**Variables**	**AKR1B10**		
	**Negative**	**Positive**	***P* value**
Age (years)			
≤60	30	27	0.551
>60	37	41	
Gender			
Male	33	24	0.863
Female	44	34	
Size (cm)			
<5	26	31	0.702
≥5	33	45	
Tumor location			
Right	19	15	0.595
Left	18	17	
Rectal	30	36	
Depth of tumor invasion			
T1-2	7	18	0.017^a^
-4	60	50	
Lymph node metastasis			
No	19	47	<0.001^b^
Yes	48	21	
Degree of differentiation			
Well	53	58	0.347
Poor	14	10	
TNM staging			
I-II	17	47	<0.001^b^
III-IV	50	21	

^a^ *P* < 0.05, ^b^ *P* < 0.001

**Table 2:** Results of univariate and multivariate analyses of postoperative patients’ survival by Cox’s proportional hazard model.

**Varieties**	**n**	**Univariate analysis**			**Multivariate analysis**		
		**HR**	**95% CI**	***P***	**HR**	**95% CI**	***P***
Age (≤60 or >60 years)	57/78	1.084	0.696-1.687	0.722			
Gender (Male / Female)	77/58	0.876	0.561-1.366	0.559			
Size of tumor (≤5 or >5 cm)	59/76	0.654	0.418-1.023	0.063			
Depth of tumor invasion (T1-2 / T3-4)	25/110	0.223	0.102-0.487	<0.001^c^	0.360	0.161-0.805	0.013^a^
Lymph node metastasis (negative / positive)	66/69	0.179	0.108-0.298	<0.001^c^	7.731	1.656-36.084	0.009^b^
Degree of differentiation (moderate-well/poor)	111/24	0.461	0.270-0.787	0.005^b^	0.799	0.457-1.395	0.429
TNM staging (I-II / III-IV)	64/71	0.157	0.093-0.264	<0.001^c^	0.033	0.006-0.164	<0.001^c^
AKR1B10 expression (negative / positive)	67/68	3.880	2.403-6.266	<0.001^c^	2.492	1.491-4.164	<0.001^c^

^a^ *P* < 0.05, ^b^ *P* < 0.01, ^c^ *P* < 0.001

### Ectopic AKR1B10 inhibits proliferation and migration of CRC cells in vitro

**Figure 2:**
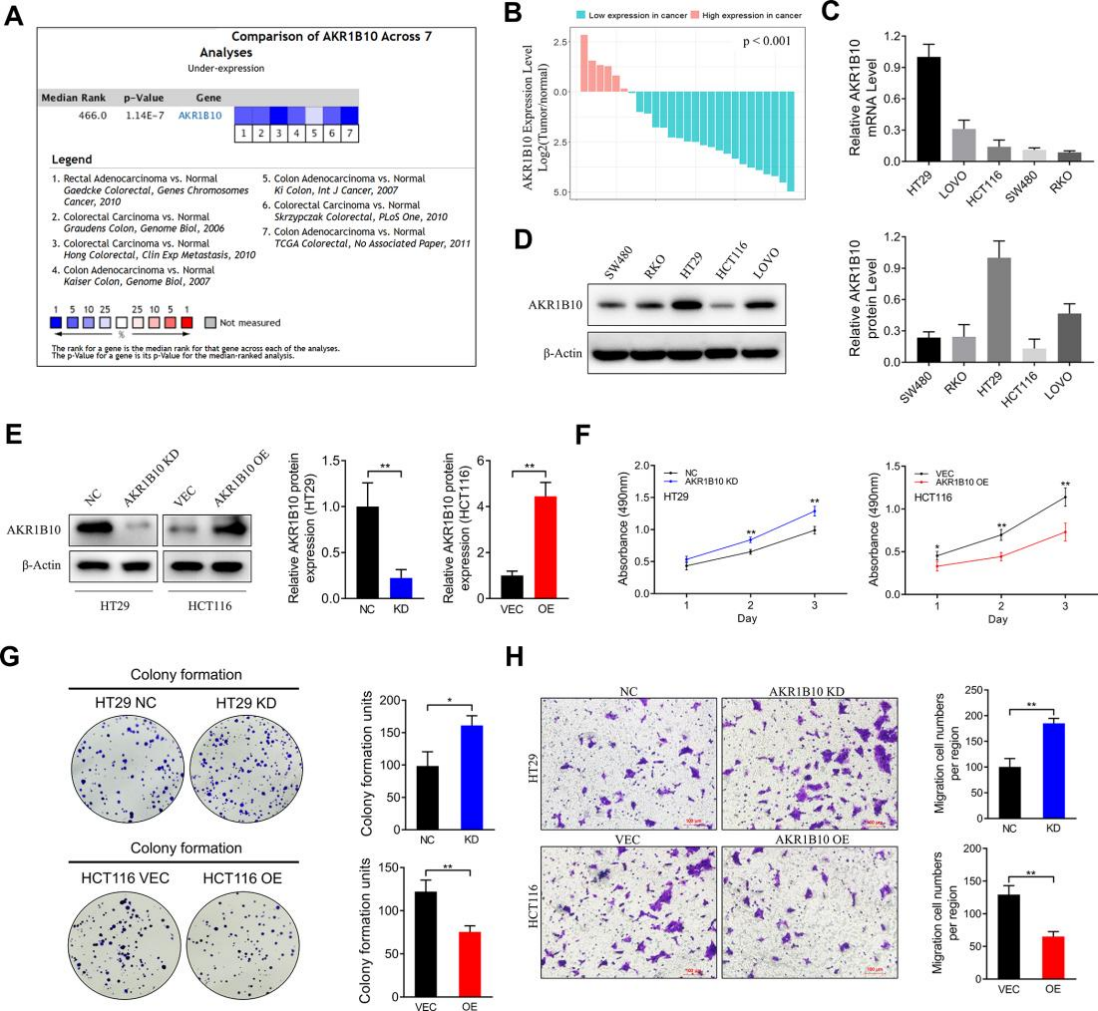
**Effect of AKR1B10 on CRC cell proliferation and migration ability.** (**A**) Comparison of AKR1B10 mRNA expression in CRC and normal tissues across 7 Oncomine datasets. (**B**–**C**) AKR1B10 mRNA levels in (**B**) 27 paired CRC and normal tissues and (**C**) 5 CRC cell lines. (**D**–**E**) Immunoblots showing AKR1B10 protein levels in (**D**) wild type and (**E**) AKR1B10-KD and AKR1B10-OE CRC cell lines. (**F**–**H**) Proliferation rates (**F**), colony forming capacity (**G**) and migration rates (**H**) of AKR1B10-KD and AKR1B10-OE CRC cells. CRC, colorectal cancer. CTL, control; NC, negative control; KD, AKR1B10-shRNA; VEC, vector; OE, AKR1B10 overexpression plasmid. Data are presented as mean ± SD (n=3). *P < 0.05, **P < 0.01, ***P < 0.001.

Pooled analysis of CRC and normal tissues across 7 Oncomine datasets (Figure 2A) revealed significant downregulation of *AKR1B10* mRNA in the CRC tissues, which was also consistent with the findings of Gaedcke et al, Kaiser et al and Hong et al (Supplementary Figure 2A). Furthermore, *AKR1B10* expression was also downregulated in the CRC tissues of our cohort compared to the paired normal tissues (Figure 2B, Supplementary Figure 2B–2C), as well as in multiple CRC cell lines (Figure 2C–2D, Supplementary Figure 2D). The HT29 cells expressed the highest levels of AKR1B10, while that in the SW480, HCT116 and RKO cells were relatively low. The biological role of AKR1B10 was further analyzed using knockdown (KD) and overexpression (OE) constructs (Figure 2E). The proliferation rate of AKR1B10-KD cells was significantly higher, and that of AKR1B10-OE cells was inhibited compared to the negative controls (Figure 2F). Consistent with this, the AKR1B10-KD cells also showed enhanced colony-formation ability, which was markedly suppressed in the AKR1B10-OE cells (Figure 2G). Overexpression of AKR1B10 also inhibited *in vitro* migration of CRC cells, whereas its knockdown had the opposite effect (Figure 2H). Taken together, AKR1B10 acts as a tumor suppressor in CRC, and its ectopic expression promotes the growth of CRC cells *in vitro*.

### AKR1B10 is closely related with FGF1 expression levels in CRC tissues

**Figure 3:**
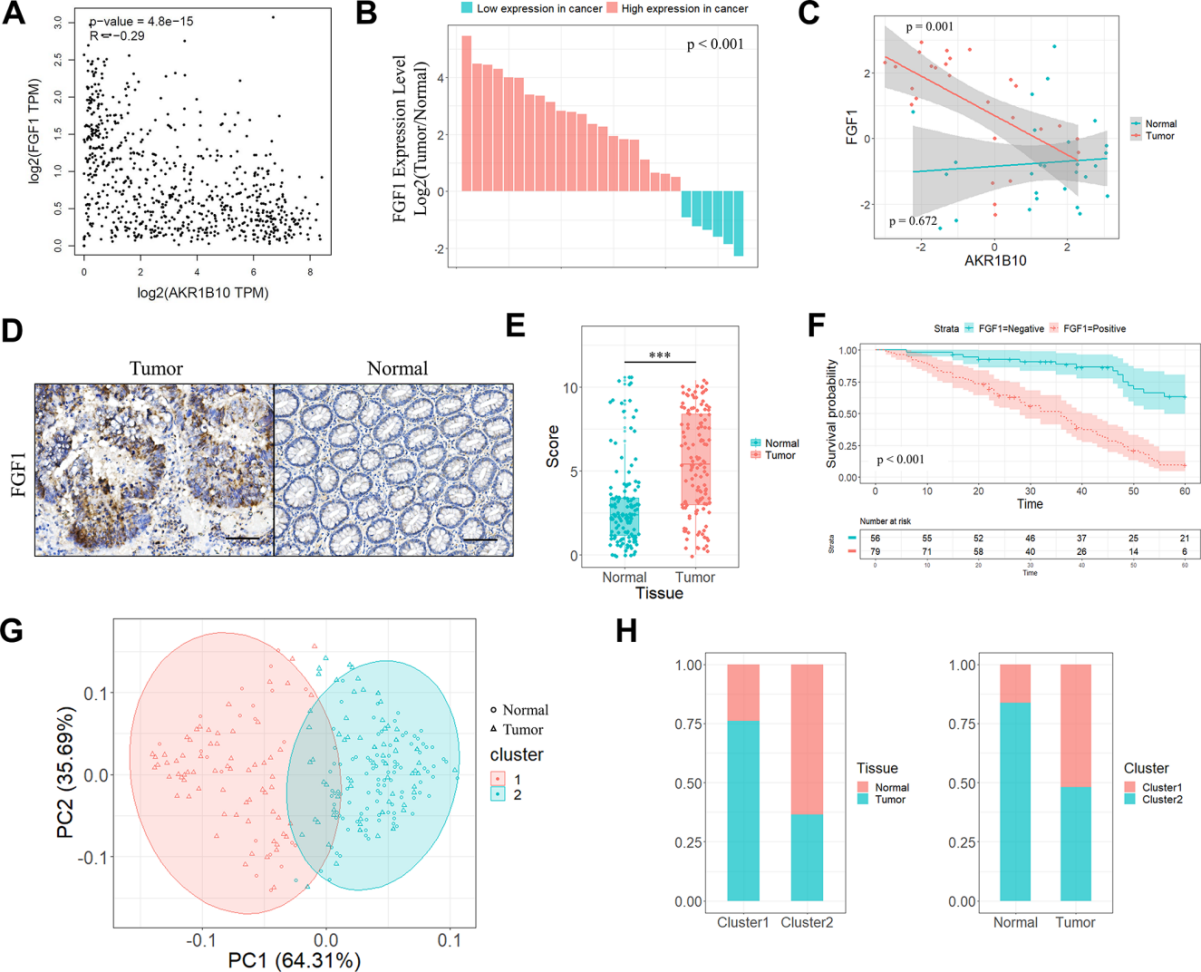
**Correlation between AKR1B10 and FGF1 in CRC tissues.** (**A**) Correlation analysis of AKR1B10 and FGF1 levels in CRC tissues from TCGA datasets by GEPIA platform. (**B**) FGF1 mRNA levels in 27 paired CRC and normal tissues. (**C**) Correlation between AKR1B10 and FGF1 levels in the above. (**D**) Representative IHC images showing *in situ* FGF1 expression in CRC and normal tissues (scale bar = 100μm) and (**E**) corresponding IHC scores. (**F**) OS of 135 CRC patients demarcated by FGF1 expression levels. (**G**) Stratification of 135 pairs of CRC and normal tissues into cluster 1 (red) and cluster 2 (green) according to AKR1B10 and FGF1 IHC scores. (**H**) Percentage of tumor and normal samples in each cluster. CRC, colorectal cancer. OS, overall survival. *** *P* < 0.001.

Since FGF1 is associated with inflammation in the tumor microenvironment, we next analyzed the potential correlation between AKR1B10 and FGF1 in TCGA datasets. AKR1B10 expression levels in the CRC tissues were closely related to that of FGF1 (*P* < 0.001, Figure 3A). Furthermore, FGF1 mRNA levels were also significantly higher in most CRC tissue specimens compared to the paired normal tissues (*P* < 0.001, Figure 3B, Supplementary Figure 3A, 3B). Interestingly, high AKR1B10 levels were significantly correlated with reduced FGF1 expression in CRC tissues (*P* = 0.001), while no such correlation was seen in normal tissues (*P* > 0.05, Figure 3C). Based on both variables, the tumor and normal groups were stratified into two clusters (Supplementary Figure 3C–3D), and most normal specimens belonged to Cluster 1 (71.4%) as opposed to Cluster 2 (28.6%) whereas the tumor samples were concentrated in Cluster 2 (63% compared to 37% in Cluster 1). The FGF1 protein levels were also significantly higher in CRC compared to the normal tissues (Figure 3D–3E), and its reduced expression was predictive of longer survival (Figure 3F). In the cluster analysis as well, the IHC scores of AKR1B10 and FGF1 were significantly different between tumor and normal tissues (Figure 3G), with 23.9% and 76.1% of the normal samples, and 51.9% and 48.1% tumor samples respectively present in Cluster 1 and Cluster 2 (Figure 3H). Taken together, AKR1B10 and FGF1 levels can distinguish between CRC and normal tissues.

### AKR1B10 inhibits colorectal tumorigenesis in vivo by targeting FGF1

**Figure 4:**
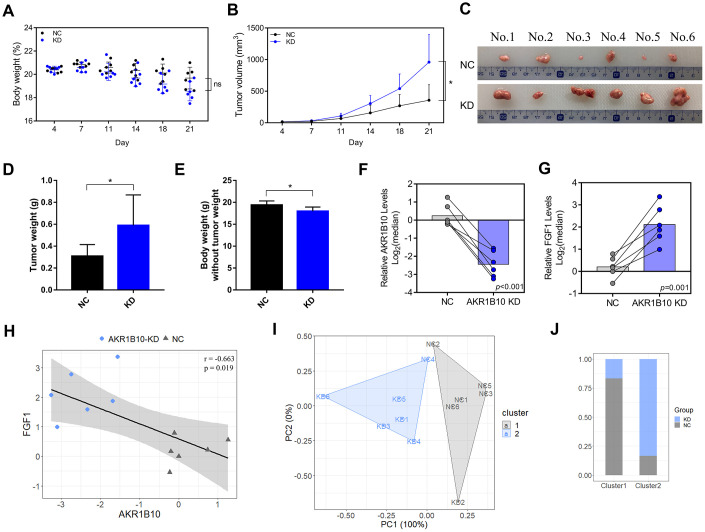
**AKR1B10 knockdown suppresses CRC tumor growth *in vivo*.** (**A**–**B**) Total body weight (**A**) and tumor volume (**B**) of the mice during the experiment. (**C**) Representative pictures of subcutaneous tumors harvested from NC and AKR1B10-KD group. (**D**) The weights of tumor masses. (**E**) Net body weight after subtracting the respective tumor weights. (**F**–**G**) Relative AKR1B10 (**F**) and FGF1 (**G**) mRNA levels in the tumors and their (**H**) correlation. (**I**) Stratification of mice into cluster 1 (grey) and cluster 2 (blue) according to body weight, tumor volume, tumor weight and AKR1B10 and FGF1 mRNA levels. (**J**) Percentage of NC and AKR1B10-KD mice in each cluster. Data are presented as mean ± SD. CRC, colorectal cancer. NC, negative control; KD, AKR1B10-shRNA. *P < 0.05, **P < 0.01, ***P < 0.001.

**Figure 5:**
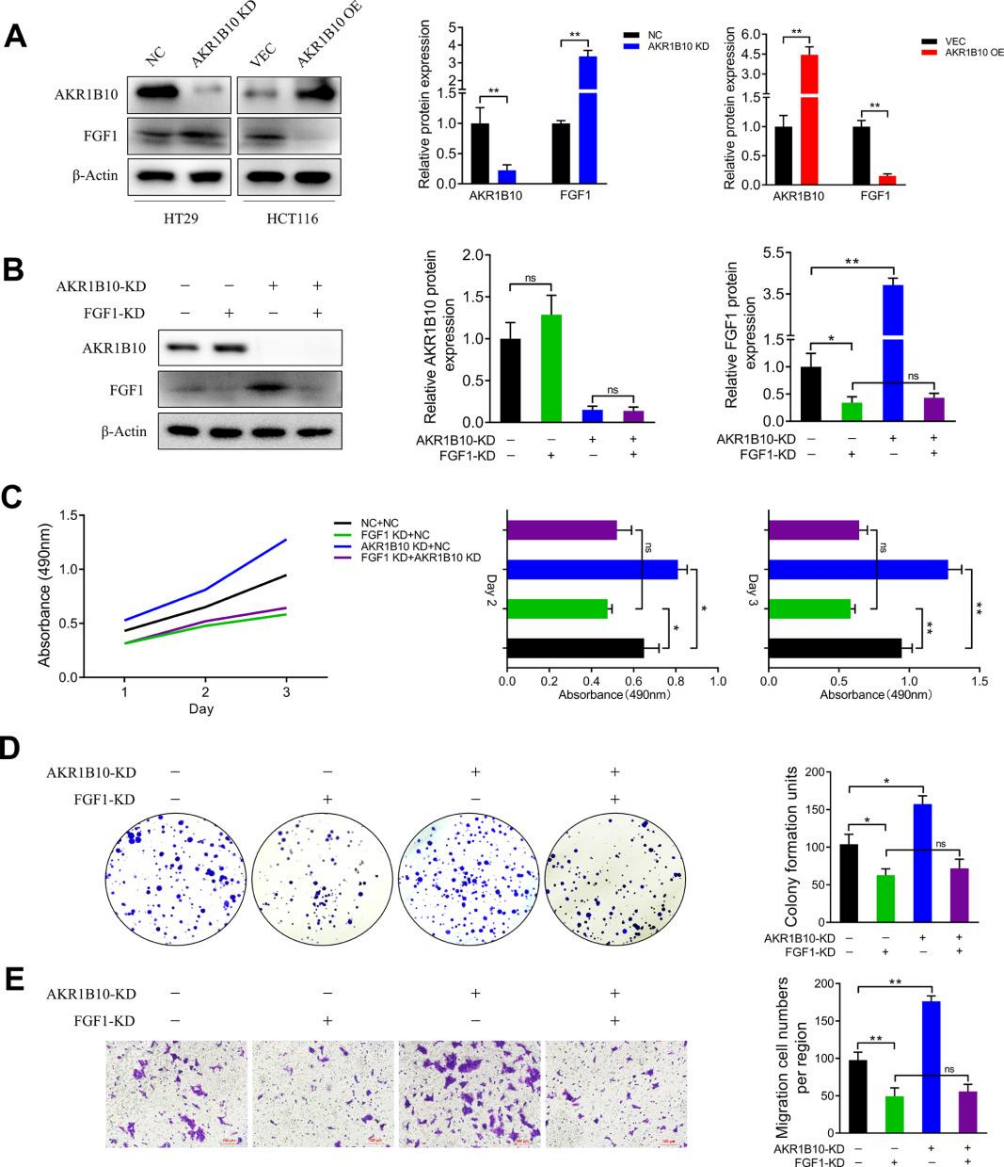
**AKR1B10 inhibits CRC cell growth in an FGF1-dependent manner.** (**A**) Immunoblot showing AKR1B10, FGF1 and GAPDH protein levels in HT29 cells transfected with AKR1B10-shRNA and in HCT116 cells transfected with AKR1B10 overexpression plasmid. (**B**) Immunoblot showing AKR1B10, FGF1 and GAPDH protein levels in HT29 transfected with FGF1-shRNA alone or in combination with AKR1B10-shRNA. (**C**–**E**) Proliferation rates (**C**), colony forming capacity (**D**) and migration rates (**E**) of the HT29 cells transfected as above. Data are presented as mean ± SD. NC, negative control; KD, AKR1B10-shRNA; VEC, vector; OE, AKR1B10 overexpression plasmid. “-”, control-shRNA. “+”, AKR1B10 or FGF1 shRNA. *P < 0.05, **P < 0.01, ***P < 0.001.

The role of AKR1B10 in CRC tumor growth was analyzed by establishing an *in vivo* xenograft model using wild-type and AKR1B10-KD HT29 cells. Depletion of AKR1B10 had no obvious effect on the body weight of the mice (Figure 4A), but significantly enhanced the proliferative capacity of the CRC cells, which was manifested as increased tumor size (Figure 4B) and weight (Figure 4C–4D) compared to control group. However, the net body weights obtained after subtracting the tumor weights were significantly lower in the mice implanted with AKR1B10-KD CRC cells (Figure 4E). Furthermore, *in situ* AKR1B10 mRNA levels were markedly lower and that of FGF1 was higher in the AKR1B10-KD tumors (Figure 4F–4G), and showed significant statistical correlation (Figure 4H). We next performed a cluster analysis to consider the combined effects of body weight, tumor volume, tumor weight and AKR1B10/FGF1 levels (Figure 4I), and found that 16.67% of the AKR1B10-KD and 83.33% of the NC group mice were in Cluster 1 (Figure 4J). To gain further mechanism insights, we analyzed the FGF1 levels in CRC cells transfected with AKR1B10-shRNA or AKR1B10 overexpression plasmid, and found that AKR1B10 downregulated FGF1 while knocking it down had the opposite effect (Figure 5A). To further determine the role of FGF1 in AKR1B10-mediated regulation of CRC progression, the HT29 cells were co-transfected with AKR1B10-shRNA and FGF1-shRNA. Interestingly, inhibiting AKR1B10 restored FGF1 expression levels following the latter’s knockdown (Figure 5B) but its overexpression did not rescue the CRC cells from the anti-proliferative effects of FGF1 knockdown (Figure 5C–5E). Taken together, AKR1B10-mediated inhibition of CRC cells is dependent on FGF1.

## DISCUSSION

AKR1B10 metabolizes various substrates such as retinaldehyde, lipid peroxidation products, and xenobiotics [5, 25–27]. It is primarily expressed in normal gastrointestinal epithelial tissues, and usually non-detectable in non-digestive tract tissues [28, 29]. Recent studies have implicated AKR1B10 in tumor growth and metastasis, and reported aberrant expression levels in various cancers [14, 30]. We found that AKR1B10 levels were high in the normal colorectal tissues and decreased significantly in primary CRC tumors compared to the surrounding normal tissues. Furthermore, CRC patients overexpressing AKR1B10 had better OS compared to the low-expressing group, which is consistent with previous studies [16, 31–33]. Nevertheless, the difference in the expression levels of AKR1B10 in the gastrointestinal and other solid tumors [12, 14–16, 34–38] has limited the clinical relevance of AKR1B10 as a therapeutic target. Although a previous study correlated AKR1B10 expression to the prognosis of CRC patients [15], its role in CRC development remains unclear. We found that reduced levels of AKR1B10 in the tumor tissues correlated significantly with advanced stages, greater invasiveness, increased tumor differentiation and poor survival of CRC patients, indicating that AKR1B10 is a potential tumor suppressor in CRC. Consistent with this, ectopic expression of AKR1B10 in the CRC cells significantly inhibited their proliferation, clonal expansion and migration *in vitro*.

AKR1B10 is a potential biomarker of CRC, although the mechanisms underlying AKR1B10 down-regulation in CRC and AKR1B10-mediated tumorigenesis remain to be clarified. Overexpression of AKR1B10 significantly inhibited the proliferation and migration of CRC cells. Correlation analysis on TGCA datasets showed a significant association between AKR1B10 and FGF1. The latter is a member of the fibroblast growth factor family that is involved in cell proliferation and migration [39–41], and acts as an oncogene in several cancers. FGF1 is aberrantly expressed in pancreatic cancer, lung cancer, glioblastoma and prostate cancer [42–45]. Elevated FGF1 levels are associated with increased angiogenesis and decreased survival in serous ovarian adenocarcinoma [46], and is a potential therapeutic target for ovarian cancer [47, 48]. We found that FGF1 was overexpressed in CRC tissues and predicted poor prognosis. Furthermore, cluster analysis indicated that both FGF1 and AKR1B10 expression levels were able to distinguish between the tumor and adjacent normal tissues, and pointed to a functional relationship as well.

AKR1B10 and AKR1B1 are closely related to inflammation [15, 19], and AKR1B10 in particular regulates inflammatory factors in the tumor microenvironment, which mobilizes the host immune response and promotes tumor suppression [15, 19, 49]. FGF1 activation is mediated via the PI3K-Akt signaling pathway that lies upstream of mTOR [50], which is related to autophagy, apoptosis and metabolism of cancer cells, as well as the NLRP3-mediated inflammatory response [51, 52]. Based on previous evidence and our findings, we hypothesized that AKR1B10 would inhibit the proliferation and migration of CRC cells by regulating FGF1-dependent signaling pathways. Indeed, AKRB110 inhibited FGF1 in CRC cell lines, and elevated FGF1 in response to AKR1B10 depletion promoted xenograft tumor growth in a mouse model. In addition, an inverse correlation between FGF1 and AKR1B10 was also observed in human CRC tumors. The likely mechanism underlying the inhibitory effect of AKR1B10 is the induction of an anti-tumor inflammatory response [15, 53] by targeting FGF1, which is related to the growth and migration of CRC cells [54, 55]. The involvement of an FGF1-dependent pathway is significant in the context of therapeutically targeting AKR1B10 in CRC [56]. Since AKR1B10 was not able to rescue CRC cells after FGF1 knockdown, the latter is possibly a downstream target of AKR1B10. Although the exact regulatory mechanism warrants future investigation, our findings provide a rationale for targeting both as a combination therapy for CRC.

## MATERIALS AND METHODS

### Human tissue specimens

A total of 135 pairs of CRC and adjacent normal colon tissues were collected immediately after surgical resection at the Department of General Surgery of the First Affiliated Hospital of Soochow University (Suzhou, China) from 2010 to 2013. None of the patients had received radiotherapy or chemotherapy before radical surgery, and all tissue specimens were verified histo-pathologically. The study was approved by the Independent Ethics Committee of the First Affiliated Hospital of Soochow University (IRB approval number, 2020-076), and all patients provided written informed consent.

### Immunohistochemistry (IHC) evaluation

Tissue specimens were fixed with 10% formalin, embedded in paraffin, and cut into 5μm-thick sections. After cleaned in xylene and rehydrated through an ethanol gradient, the sections were treated with 3% hydrogen peroxide to quench endogenous peroxidases, and then boiled in 10mM citrate buffer (pH 6) for antigen retrieval. The processed sections were then blocked with 10% goat serum for 30 min, and incubated overnight with 1:200 diluted polyclonal anti-human AKR1B10 (BOSTER, Wuhan, China) or anti-human FGF1 (BOSTER, Wuhan, China) at 4°C. Color was developed using a tissue staining kit (Zhongshan Biotechnology, Beijing, China). The AKR1B10 or FGF1 staining scores were evaluated in five random fields per slide by two pathologists YuHong Wang (The First Affiliated Hospital of Soochow University) and Zheng Zhi (The Soochow University) in a blinded manner as previously described [24]. The percentage of positively stained cells was scored as follows: 0 - 0-5%; 1 - 6-25%; 2 - 26-50%; 3 - 51-75%; 4 - >75%. The staining intensity was scored as 0 (negative), 1 (weak), 2 (moderate) and 3 (strong). The final score was the average of the percentage score multiplied by intensity score, and graded as follows: – (0), + (1-4), ++ (5-8) and +++ (9-12). Samples with final scores ++ or +++ were graded as positive, and – or + as negative.

### Bioinformatics analysis

CRC gene expression datasets were downloaded from the Oncomine (https://www.oncomine.org), CCLE (Cancer Cell Line Encyclopedia, https://portals.broadinstitute.org/ccle) and GEPIA (Gene Expression Profiling Interactive Analysis, http://gepia.cancer-pku.cn) databases, and analyzed by established protocols.

### Survival analysis

All patients were followed up by personal or telephonic interviews for 60 months, and the time point was set as the date of CRC-related death or 60 months after surgery. Self-developed R program (version 3.6.1 for Windows, http://cran.r-project.org/) was used for sample classification and prognostic analysis. The patients were classified into two subgroups according to the IHC staining scores, and Kaplan-Meier survival curves were plotted for both groups using the “survminer” package (version 0.4.6, https://cran.r-project.org/web/packages/survminer/index.html). The log-rank test was used for statistical comparison and *P* < 0.05 was considered significant.

### Cell culture and transfection

Five human CRC cell lines (HCT116, HT29, LOVO, SW480 and RKO) were purchased from the Cell Bank of Chinese Academy of Sciences (Shanghai, China), and were cultured in RPMI 1640 medium (Hyclone) supplemented with 10% fetal bovine serum (Gibco, USA), penicillin G sodium (100U/ml) and streptomycin (100μg/ml) at 37°C under 5% CO2. The HT29 cells were grown till 70% confluency, and transfected with human AKR1B10 or human FGF1 shRNA according to the manufacturer’s instructions. The transfected cells were selected using 500μg/ml G418 (Roche, Switzerland) for 3-4 weeks, and clones with a stable knockdown of AKR1B10 or FGF1 were selected for further experiments. Control cells were stably transfected with scrambled shRNA. In addition, 70% confluent HCT116 cells were transfected with the AKR1B10 cDNA or empty plasmid using X-tremegene HP at 1:1 ratio, and harvested after 24h. Transient overexpression and silencing were confirmed by RT-PCR and Western blotting. All stable transfectants were used by the 8^th^ passage.

### RNA isolation and quantitative real-time PCR (qRT-PCR)

Total RNA was extracted from the tissues or cells using TRIzol reagent (Invitrogen, Life Technologies, USA) according to the manufacturer’s protocol. Following DNAse I (Thermo Fisher Scientific, USA) treatment to remove genomic DNA, 1μg RNA was reverse transcribed using a RevertAid First Strand cDNA Synthesis Kit (Thermo Fisher Scientific, USA). The qRT-PCR was performed using Power SYBR® Green PCR Master Mix (ABI, USA) on the 7500 real time PCR system (ABI, USA) according to the manufacturer’s instructions. Fold changes were calculated relative to β-actin (internal control) using the 2^-ΔΔC^T method. The following primers were used: AKR1B10 forward (5’-CCCAAAGATGATAAAGGTAATGCCATCGGT-3’) and reverse (5’-CGATCTGGAAGTGGCTGAAATTGGAGA-3’); FGF1 forward (5’-GTGGATGGGACAAGGGACAG-3’) and reverse (5’-GGCAGGGGGAGAAACAAGAT-3’); β-actin forward (5’- CCACACTGTGCCCATCTACG-3’) and reverse (5’-AGGATCTTCATGAGGTAGTCAGTCAG-3’). The PCR conditions were: initial denaturation at 95°C for 5 min, followed by 40 cycles of denaturation at 95°C for 30 sec, annealing at 55°C for 30 sec and extension at 72°C for 30 sec, and final extension at 72°C for 7 min.

### Protein isolation and Western blotting

Cells were lysed in ice-cold RIPA lysis buffer supplemented with protease and phosphatase inhibitors (KeyGEN Inc., Nanjing, China) according to the manufacturer’s protocol. The extracted proteins were separated by SDS-PAGE and transferred onto PVDF membranes (Millipore, USA). After blocking with 5% non-fat milk for 1h, the membranes were probed overnight with anti-AKR1B1 (1:1000, Cell Signaling Technology), anti-FGF1 (1:1000, Cell Signaling Technology) and anti-β-Actin (1:5000, Cell Signaling Technology) antibodies at 4°C with gentle shaking, followed by horseradish peroxidase-conjugated secondary antibodies. The protein bands were visualized by chemiluminescence and quantified by ImageJ for Windows (NIH, USA).

### MTT assay

Cell viability was determined using an MTT assay kit (Amresco, USA) according to the manufacturer’s instructions. Briefly, 2000 transfected cells were seeded in 96-well plates, and cultured for 12, 24, 36, 48, 60 and 72h. The MTT solution was added 4h prior to the termination of each time point, and the supernatants were removed. The formazan crystals were dissolved in 150μl DMSO per well for 10 min with gentle shaking, and the absorbance at 490nm was measured using a microplate reader.

### Cell migration assay

Cell migration was assessed using Transwell inserts (pore size 8μm; Corning, New York, USA). The cells were seeded into the upper chambers of the inserts at the density of 50,000 cells/200μl in serum-free RPMI 1640 medium, and the lower chambers were filled with 750μl complete medium per well. After incubating for 24h at 37°C, the cells remaining on the upper surface of the membrane were removed using a cotton swab. The filters were then fixed with 4% paraformaldehyde, and the cells on the lower surface were stained with 0.1% crystal violet and counted in 5 random fields per sample.

### Colony formation assay

The suitably transfected cells were seeded in 6-well plates at the density of 1000 cells/well, and cultured for 10 days before being fixed and stained with 0.1% crystal violet. The colonies with more than 100 cells were counted at 40x magnification under an optical microscope (Nikon, Japan) fitted with a digital camera (Nikon, Japan).

### Subcutaneous xenograft establishment

SPF male BALB/c nude mice (3-5weeks old and weighing 16-18 g) were purchased from Shanghai SLRC laboratory Animal Co. Ltd. (Shanghai, China). The mice were randomly divided into the AKR1B10 knock down (KD) and negative control (NC) groups (n = 6 per group), and accordingly injected subcutaneously with 5×10^6^ AKR1B10-KD or NC-shRNA HT29 into the left and right dorsal flank on day 0. All animal experiments were approved by the Animal Ethics Committee of Soochow University (Suzhou, China).

### Statistical analysis

All data were presented as mean ± SD of three independent experiments. Statistical analyses were performed using SPSS 22.0 software (SPSS Inc., Chicago, IL, USA), GraphPad Prism 8 (San Diego, CA) and R programs. The Student's t-test (unpaired, two-tailed), Mann–Whitney *U* test or one-way ANOVA were used to compare means between groups. IHC results were analyzed by Chi-squared or Fisher's exact tests. Unsupervised learning cluster analysis was performed using the “cluster” package (version 2.1.0, https://cran.r-project.org/web/packages/cluster/index.html) in R programs. *P* < 0.05 was considered statistically significant.

## Supplementary Material

Supplementary Figures
