# Supplementary material for: Loss of AKR1B10 promotes colorectal cancer cells proliferation and migration via regulating FGF1-dependent pathway
Source: Aging (Albany NY). 2020 Jul 2;12(13):13059–75. doi: 10.18632/aging.103393 (PMC7377871; doi:10.18632/aging.103393)
Supplement: Supplementary Figures [file aging-12-103393-s001..pdf]

SUPPLEMENTARY FIGURES

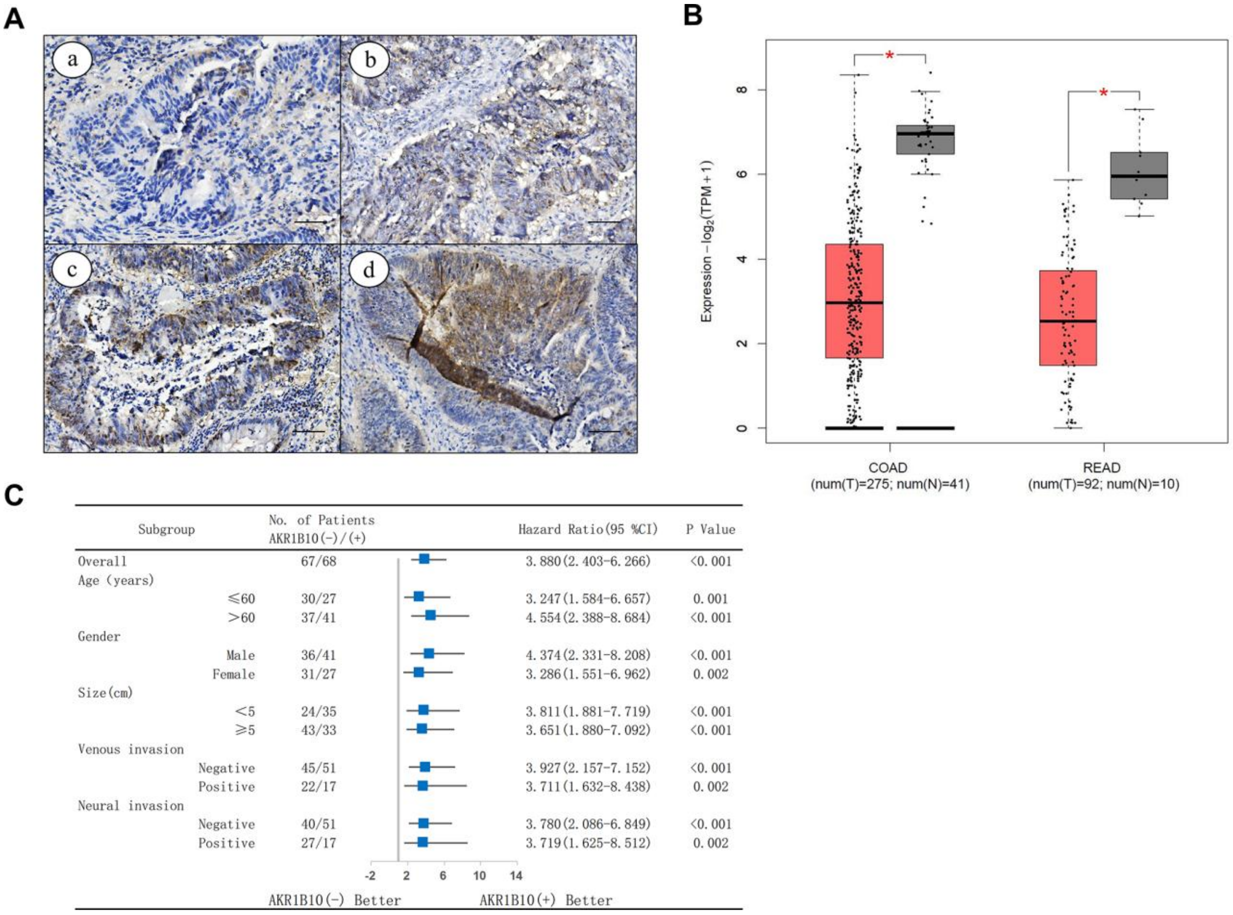

Supplementary Figure 1. Expression of AKR1B10 in CRC in TCGA datasets. (**A**) IHC images showing *in situ* AKR1B10 expression in CRC tissues (scale bar = 100μm). Negative (a), weak (b), positive (c), strong positive (d). (**B**) Comparison of AKR1B10 levels between CRC and paired normal tissues in TCGA datasets by GEPIA platform. (**C**) OS of AKR1B10^{pos} and AKR1B10^{neg} CRC patients in the subgroups of age, gender, tumor size, venous invasion and neural invasion. CRC, colorectal cancer. * $P < 0.05$.

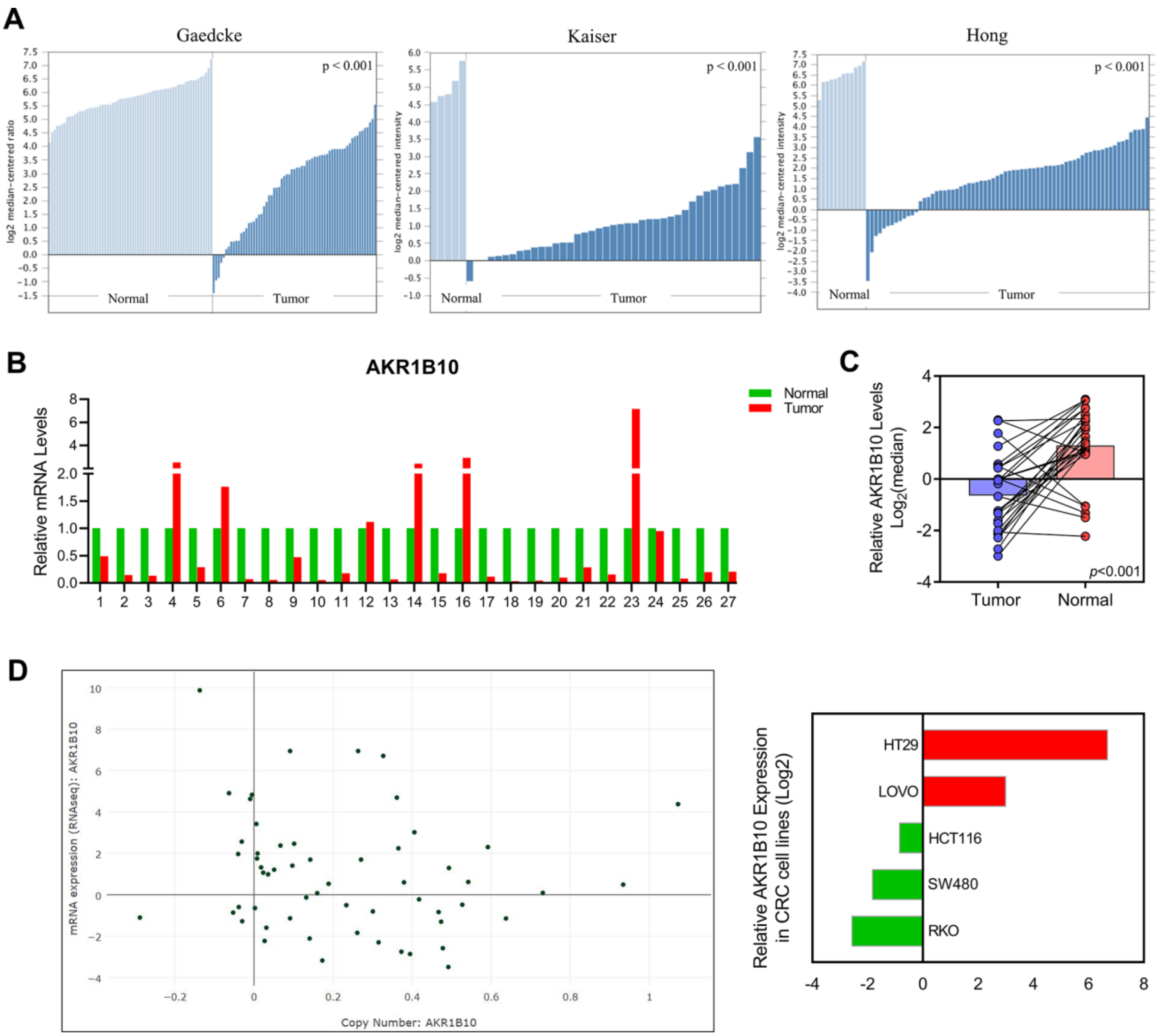

Supplementary Figure 2. AKR1B10 expression in CRC tissues and cell lines. (**A**–**B**) AKR1B10 mRNA levels in (**A**) CRC and non-tumor tissues in Oncomine datasets and (**B**) 27 paired CRC and normal tissues. (**C**) Relative AKR1B10 expression in 27 paired CRC and normal tissues. (**D**) AKR1B10 expression in 5 CRC cell lines from the CCLE platform. CRC, colorectal cancer. CCLE, Cancer Cell Line Encyclopedia.

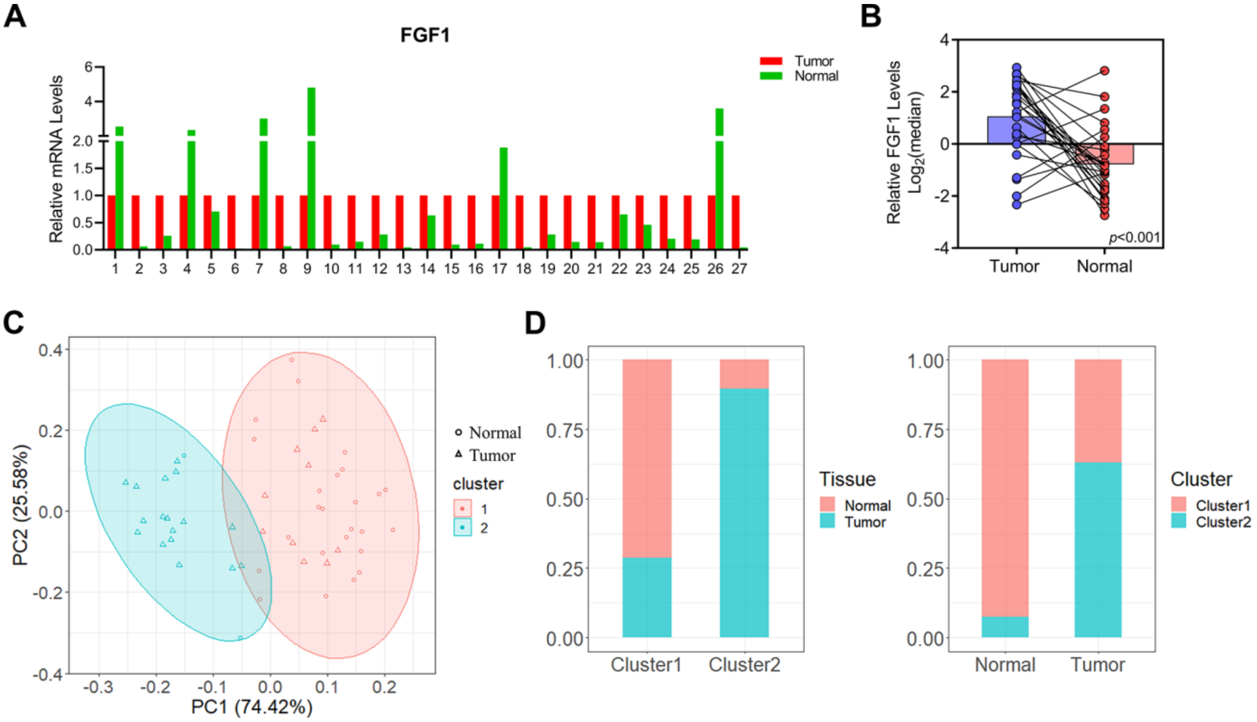

Supplementary Figure 3. FGF1 expression in CRC and paired normal tissues. (**A**) FGF1 mRNA levels in 27 paired CRC and normal tissues and (**B**) the relative expression levels. (**C**) Stratification of the 27 pairs of CRC and normal tissues into cluster 1 (red) and cluster 2 (green) according to AKR1B10 and FGF1 mRNA levels. (**D**) Percentage of tumor and normal samples in each cluster. CRC, colorectal cancer.
